# Development of Hexaploid Wheat Germplasm with Resistance to Both Powdery Mildew and Stripe Rust by Introgression of *Pm60* and *YrU1* from *Triticum urartu*

**DOI:** 10.3390/plants15121802

**Published:** 2026-06-11

**Authors:** Wei Pan, Jingyuan Yang, Boyuan Zhang, Jiarui Zhang, Junna Sun, Zuhuan Yang, Nannan Liu, Wenxin Wei, Qiang Zhang, Tzion Fahima, Weilong Guo, Jun Ma, Yinghui Li, Chaojie Xie

**Affiliations:** 1 State Key Laboratory of High-Efficiency Production of Wheat-Maize Double Cropping, Frontiers Science Center for Molecular Design Breeding, Key Laboratory of Crop Heterosis and Utilization (MOE), Beijing Key Laboratory of Crop Genetic Improvement, China Agricultural University, Beijing 100193, China; panw2023@163.com (W.P.);; 2 Triticeae Research Institute, Sichuan Agricultural University, Chengdu 611130, China; y13233088533@163.com; 3 State Key Laboratory of Crop Gene Exploration and Utilization in Southwest China, Sichuan Agricultural University, Chengdu 611130, China; 4 State Key Laboratory of Seed Innovation, Instiute of Genetics and Developmental Bidlogy, Chinese Academy of Sciences, Beijing 100101, China; 5 The Industrial Crop Institute, Shanxi Agricultural University, Taiyuan 030031, China; 6 Department of Evolutionary and Environmental Biology and the Institute of Evolution, University of Haifa, Mt. Carmel, Haifa 3498838, Israel

**Keywords:** *Triticum urartu*, synthetic amphidiploid wheat, *Pm60*, *Pm60b*, *YrU1*, marker-assisted selection, powdery mildew, stripe rust

## Abstract

Wheat powdery mildew and stripe rust, caused by *Blumeria graminis* f. sp. *tritici* (*Bgt*) and *Puccinia striiformis* f. sp. *tritici* (*Pst*), respectively, are two devastating diseases that threaten global wheat production. Long-term reliance on a limited number of resistance genes can accelerate resistance breakdown. *Triticum urartu* (2*n* = 14, A^u^A^u^), the progenitor of the wheat A subgenome, serves as a valuable gene pool for disease resistance. In this study, we identified three *T. urartu* accessions exhibiting high resistance to *Bgt* and *Pst*. Molecular marker analysis indicated that PI 428215 and PI 428315 carry *Pm60b*, whereas CITR 17664 carries both *Pm60* and *YrU1*. Durum–*T. urartu* amphiploids (AABBA^u^A^u^) displayed resistance responses identical to their *T. urartu* parent and were used as bridges to transfer these resistance genes into a common wheat (AABBDD) background. Using marker-assisted selection (MAS), recurrent backcrossing, selfing, and phenotypic screening, we developed wheat lines carrying *Pm60*, *Pm60b*, *YrU1*, or *Pm60* + *YrU1*. Segregation analysis in backcross-derived populations supported the functionality of these genes in the common wheat background. The selected introgression lines have high resistance to *Bgt* and *Pst* and showed no obvious adverse agronomic effects, providing useful germplasm for wheat disease resistance breeding. This study used a “multi-resistance, multi-combination” pyramiding strategy by MAS to introduce resistance genes from wild wheat into common wheat.

## 1. Introduction

Common wheat (*Triticum aestivum* L.) is a staple food for more than one-third of the global population, making its stable production critical to global food security [1]. However, wheat production is severely threatened by fungal diseases, including powdery mildew, caused by *Blumeria graminis* f. sp. *tritici* (*Bgt*), and stripe rust, caused by *Puccinia striiformis* f. sp. *tritici* (*Pst*). Epidemics of these diseases can cause yield losses of 10–50%, and under severe conditions, may result in complete crop failure [2,3]. Deployment of resistance genes in wheat cultivars is the most economical, effective, and environmentally friendly strategy for disease control. Nevertheless, the ongoing evolution and virulence shifts within pathogen populations frequently compromise the effectiveness of widely deployed resistance genes. The powdery mildew resistance gene *Pm3* and its alleles have largely failed due to rapid pathogen evolution [4]. Similarly, the wheat cultivar Bima 1, widely grown in China in the 1950s, lost stripe rust resistance after the emergence of race CYR18, which overcame *Yr1* [5]. These cases show that long-term reliance on a limited number of resistance genes can lead to resistance breakdown. Therefore, the discovery, introgression, and deployment of more effective resistance genes are critical to wheat disease management.

Wild relatives of wheat harbor extensive genetic diversity and represent important reservoirs of disease resistance genes. *Triticum urartu* (genome A^u^A^u^), the progenitor of the A subgenome of common wheat [6], is an especially valuable germplasm resource for wheat improvement. In recent years, several resistance genes have been cloned from *T. urartu*, demonstrating its breeding potential. For example, Zou et al. isolated the powdery mildew resistance gene *Pm60* from the *T. urartu* accession PI 428309 [7]. *Pm60* encodes a coiled-coil nucleotide-binding site leucine-rich repeat (CC-NBS-LRR, CNL) protein that confers strong resistance to the *Bgt* isolate E09. Further haplotype analysis revealed multiple functional alleles at the *Pm60* locus, with *Pm60b* showing a resistance spectrum distinct from that of *Pm60* [8]. The stripe rust resistance gene *YrU1*, cloned from the same donor *T. urartu* accession PI 428309 [9], encodes a structurally unique CNL protein that harbors an N-terminal ankyrin-repeat (ANK) domain and a C-terminal WRKY domain. Resistance evaluations showed that *YrU1* confers effective resistance against multiple prevalent *Pst* races in China, including CYR32 and CYR33. However, *YrU1* has not been introgressed into a common wheat background, which limits its exploitation in wheat breeding.

Genes derived from *T. urartu* cannot be directly utilized in breeding because crossing with common wheat is often hindered by post-pollination reproductive barriers, making it difficult to obtain fertile offspring. To overcome this challenge, researchers have developed two main introgression strategies. For example, Zou et al. introgressed *Pm60* from *T. urartu* into the common wheat cultivars Chinese Spring and Mingxian 169 using optimized immature embryo rescue technique combined with successive backcrossing. Zhang et al. proposed a “durum as a bridge” strategy and achieved the introgression of *Pm60* into hexaploid wheat with backcrossing [10]. The feasibility of such introgression strategies was demonstrated in earlier studies that transferred stem rust resistance genes *Sr21* [11], *Sr22* [12], and *Sr35* [13] as well as leaf rust resistance gene *Lr63* [14] from *Triticum monococcum*, another A-genome donor, into common wheat. However, following alien gene introgression through distant hybridization, large donor chromosomal segments may flank the target gene, a phenomenon known as linkage drag, and these segments may carry deleterious alleles affecting agronomic traits. For instance, the *Pm13* [15] from *Aegilops longissima* was transferred into wheat over 30 years ago but has not been widely utilized in breeding programs, largely due to linkage drag associated with its introgressed segment [16]. Therefore, developing resistant germplasm with acceptable agronomic performance requires repeated backcrossing, selfing, and combined genotypic and phenotypic selection.

Pyramiding multiple resistance genes into one cultivar provides simultaneous resistance to multiple diseases and reduces the use of fungicides targeting those diseases [17]. This approach is therefore important for green and efficient breeding. The elite wheat cultivar Guinong 29 pyramids powdery mildew resistance genes *Pm2* and *Pm21* with the stripe rust resistance gene *Yr26* and adult-plant slow-rusting genes for leaf rust, achieving synergistic improvement in multi-disease resistance together with superior agronomic and yield traits [18], thereby reducing the need for repeated fungicide applications. However, conventional breeding methods are inefficient for selecting individuals carrying multiple target genes. Marker-assisted selection (MAS) overcomes these limitations by enabling accurate genotyping of breeding materials in early generations and precise tracking of target gene introgression and pyramiding, thereby improving breeding efficiency. Integrating MAS with speed breeding, for example, reduced the time required to develop BC_2_ near-isogenic lines by 53% while achieving more than 91.5% recovery of the recurrent-parent genome [19].

In this study, using synthetic amphidiploid wheat (SAW) lines as a bridge, we transferred the powdery mildew resistance genes *Pm60* and *Pm60b* and the stripe rust resistance gene *YrU1* from multiple *T. urartu* accessions into hexaploid wheat. Through successive crossing, MAS, selfing, disease resistance evaluation, fertility screening, and field assessment, we obtained stable introgression lines carrying different gene combinations (*Pm60*, *Pm60b*, *YrU1*, and *Pm60* + *YrU1*). These lines showed strong resistance and no obvious adverse agronomic effects under the tested conditions, providing valuable materials for wheat disease resistance breeding.

## 2. Materials and Methods

### 2.1. Plant Materials

The original donors of the resistance genes used in this study were the *T. urartu* accessions CITR 17664 (Baal Bek-Bashari, Lebanon), PI 428215 (Mardin, Turkey), and PI 428315 (Baal Bek-Bashari, Lebanon) (Table S1). The hexaploid wheat varieties Xuezao and Fielder, both susceptible to powdery mildew and stripe rust, were used as recurrent parents for trait improvement. All *T. urartu* and hexaploid wheat materials used in this study were provided and maintained by the Wheat Research Center of China Agricultural University.

### 2.2. Breeding Pipeline

**Figure 1:**
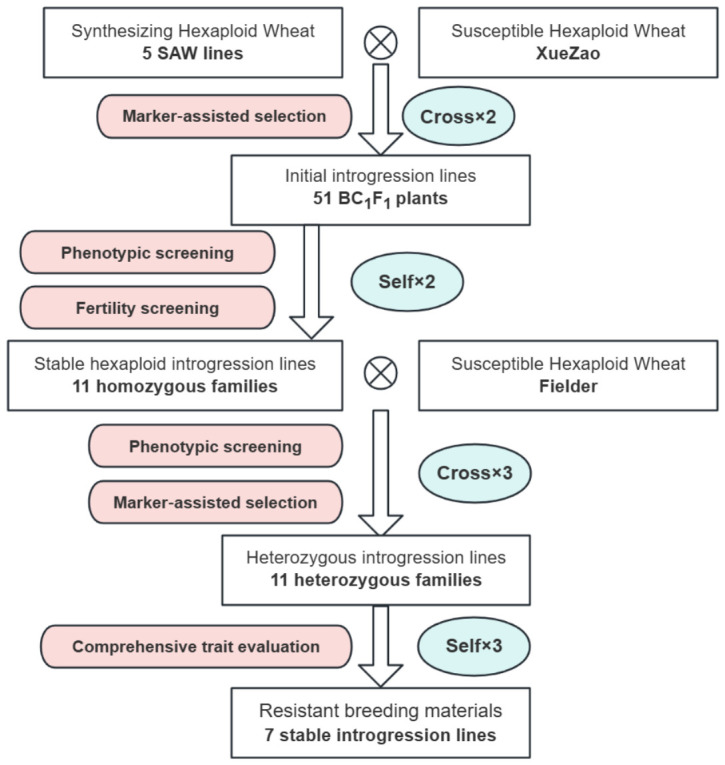
Development and screening pipeline of resistant breeding materials.

The breeding pipeline for developing resistant breeding materials in this study is illustrated in Figure 1. Five synthetic amphidiploid wheat lines (SAW1, SAW2, SAW9, SAW10, and SAW14) carrying the target resistance genes were used as donor parents. After two successive crosses with the susceptible hexaploid wheat line XueZao, combined with MAS, BC_1_F_1_ initial introgression lines were obtained. Selected plants were then selfed for two generations, accompanied by disease resistance evaluation and fertility screening, resulting in stable hexaploid introgression lines (including 1P-3, 1P-5, 2P-1, 3P-3, etc.). To further improve agronomic traits, these stable introgression lines were backcrossed three times with the spring wheat cultivar Fielder, with MAS and phenotypic screening at each generation, generating BC_2_F_1_ heterozygous introgression lines. Finally, BC_2_F_1_ plants were selfed for three generations and subjected to comprehensive trait evaluation, leading to the selection of resistant breeding materials.

### 2.3. Molecular Marker Analysis

Total genomic DNA was extracted from leaf tissue using the CTAB protocol. At each generation, the resistance genes were tracked using the markers *M-Pm60* [7] and *M-YrU1* [9] (primer sequences are listed in Supplementary Table S3). The amplified fragment of *YrU1* is 1738 bp, that of *Pm60* is 1551 bp, and that of *Pm60b* is 1791 bp. PCR was performed in a 20 μL reaction mixture containing 10 μL of 2× Taq PCR StarMix (GenStar, Beijing, China), 1 μL of DNA template (50–100 ng/μL), 2 μL of primer mix (forward and reverse, 2 μM each), and 7 μL of ddH_2_O. The amplification program was as follows: initial denaturation at 94 °C for 5 min, 34 cycles of denaturation at 94 °C for 30 s, annealing at 58 °C for 30 s, and extension at 72 °C for 1 min 30 s, followed by a final extension at 72 °C for 5 min. The PCR products were separated on 1% agarose gels by electrophoresis at a constant voltage of 200 V for 8 min.

### 2.4. Evaluation of Powdery Mildew Resistance

The powdery mildew race E09, which has been maintained in the pathogen identification greenhouse of the Wheat Research Center at China Agricultural University, was used for all powdery mildew resistance assessments in this study. All seedlings to be inoculated were grown under uniform greenhouse conditions. At the two-leaf stage, seedlings were continuously inoculated with race E09 by the dusting method using spores propagated on susceptible XueZao plants [20]. Disease responses were evaluated 15 days after inoculation using a 0–4 infection-type (IT) scale: IT 0, immune; IT 0; necrotic flecks; IT 1, high resistance; IT 2, moderate resistance; IT 3, moderate susceptibility; IT 4, high susceptibility [21]. The adult-plant resistance to wheat powdery mildew was evaluated at the Shangzhuang Experimental Station of China Agricultural University. Natural disease pressure was provided by spreader rows of XueZao inoculated with *Bgt* E09, while uninoculated XueZao served as the susceptible control. Disease assessments were performed multiple times from heading through grain filling, and disease responses were recorded using the same procedure described above.

### 2.5. Evaluation of Stripe Rust Resistance

The stripe rust race CYR34 was used in all seedling-stage stripe rust resistance evaluations. Seedlings of the test materials were grown under uniform greenhouse conditions. When the plants reached the two- to five-leaf stage, they were inoculated with an aqueous suspension of urediniospores (collected from the susceptible cultivar MX169) in isododecane (4:1, *v*/*v*) using a pneumatic spray gun. After inoculation, plants were placed in a dew chamber at 10 °C in the dark for 24 h and then transferred to two growth cabinets with different temperature regimes: a low-temperature treatment (10–15 °C) and a high-temperature treatment (10–30 °C). Both cabinets were set to a 16 h light/8 h dark photoperiod, with temperature gradually increasing from the minimum at 2:00 a.m. to the maximum at 2:00 p.m. Infection types (IT) were recorded 18–21 days post-inoculation using a 0–9 scale [22]. Adult-plant resistance to stripe rust was evaluated at the Wenjiang Experimental Station of Sichuan Agricultural University. Spreader rows of the highly susceptible cultivar XueZao were planted every 20–30 rows and around the plots to ensure uniform inoculum distribution. The spreader rows were artificially inoculated with a mixture of prevalent *Pst* races (CYR32, CYR33, and CYR34) at the tillering to stem elongation stage. Disease assessments were performed multiple times at different stages, from heading to grain filling, at 7–10-day intervals, and the final assessment was conducted when the susceptible control XueZao showed maximum disease severity (≥80% on flag leaves).

### 2.6. Determination and Calculation of Selfed Seed Set per Plant

Prior to flowering, the main stem spike or a representative tiller spike of each test plant was bagged with a parchment paper bag (or cellophane bag) to prevent cross-pollination by foreign pollen, allowing natural self-pollination. One to two spikes per plant were bagged. After grain maturity, the bagged spikes were harvested. The total number of florets (after removing the two underdeveloped spikelets at the base and the two at the tip, the sum of the two basal florets per remaining spikelet was counted) and the number of filled grains were recorded. The selfed seed set percentage was calculated using the following formula:

Selfed seed set (%) = (Number of filled grains/Total number of florets) × 100%

The average seed set percentage of 2–3 spikes per plant was taken as the selfed seed set for that plant. In this study, plants with selfed seed set ≥50% were considered to have normal fertility and were retained for subsequent generations; those with <50% selfed seed set were discarded.

### 2.7. Agronomic Trait Evaluation

Agronomic trait evaluation of hexaploid introgression lines was conducted during the 2024–2025 growing season at the Shangzhuang Experimental Station of China Agricultural University in Beijing. For each line, 15 seeds were uniformly sown in rows of 1.5 m in length with 0.3 m spacing. At maturity, 4–5 plants were randomly selected from each line, and morphological traits were evaluated, including plant height, tiller number, spike length, number of spikelets per spike, number of grains per spike, and thousand-grain weight. Statistical analysis was performed to compare the agronomic traits of each introgression line with the control Fielder. Student’s *t*-test was applied to assess the significance of differences, and *p*-values < 0.05 were considered statistically significant. The results are presented as mean ± standard deviation (SD).

## 3. Results

### 3.1. Resistance Evaluation of T. urartu and Development of Synthetic Amphidiploid Wheat

**Figure 2:**
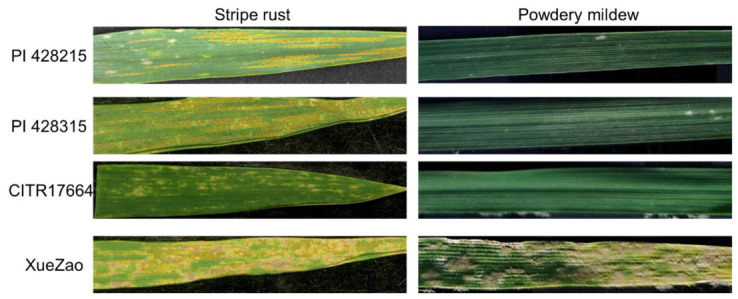
Adult-stage evaluation of stripe rust and powdery mildew resistance in *T. urartu* accessions PI 428215, PI 428315, and CITR 17664. Mixed races of *Pst* (CYR32, CYR33, CYR34) and *Bgt* E09 were used under field conditions.

**Figure 3:**
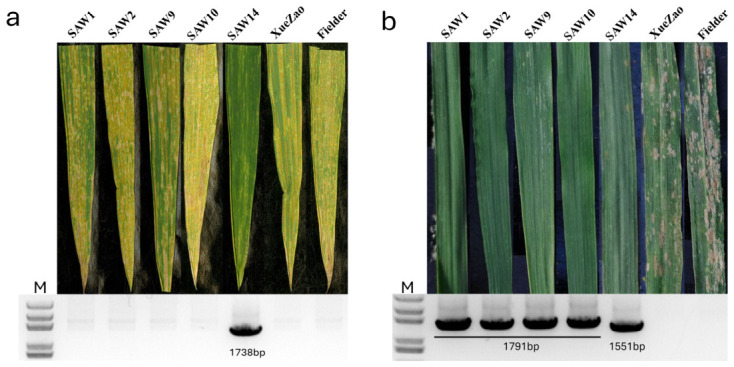
Resistance evaluation and marker detection of synthetic hexaploid wheat. (**a**) Adult-stage response of synthetic hexaploid wheat lines and recurrent parents to mixed races of stripe rust (CYR32, CYR33, and CYR34); SAW14 (highly resistant); SAW1, SAW2, SAW9, SAW10, XueZao, and Fielder (highly susceptible). (**b**) Adult-stage response of synthetic hexaploid wheat lines and recurrent parents to *Bgt* E09 of powdery mildew (*Bgt*); SAW1, SAW2, SAW9, SAW10, and SAW14 (highly resistant); XueZao and Fielder (highly susceptible); M, marker; expected fragment sizes: 1738 bp (*YrU1*), 1791 bp (*Pm60b*), and 1551 bp (*Pm60*).

Zhao et al. reported that *T. urartu* accession CITR 17664 carries *Pm60*, while PI 428215 and PI 428315 carry *Pm60b* [23]. Using the functional marker *M-YrU1* of the stripe rust resistance gene *YrU1*, we detected that CITR 17664 also carries *YrU1*. Resistance evaluation showed that PI 428215, PI 428315, and CITR 17664 were resistant to powdery mildew, and CITR 17664 was also resistant to stripe rust (Figure 2). Previously, Zhang et al. used the durum wheat (AABB) cultivar Mo75 as a bridge parent [10]. The powdery mildew-resistant *T. urartu* accessions CITR 17664, PI 428215, and PI 428315 were used as male parents to pollinate Mo75. The resulting hybrid progenies were treated with colchicine, generating a series of synthetic amphidiploid wheat (SAW) lines (AABBA^u^A^u^, 2n = 6x = 42), which can serve as bridging lines to introgress desirable genes from *T. urartu* into cultivated wheat. Five SAW lines, SAW1, SAW2, SAW9, SAW10, and SAW14 with high seed set, were selected as donor materials (Table S2). Phenotyping results showed that SAW1, SAW2, SAW9, SAW10, and SAW14 all exhibited immunity-level resistance to powdery mildew *Bgt* E09, and SAW14 also displayed immunity-level resistance to stripe rust (mixed races CYR32, CYR33, and CYR34) (Figure 3).

### 3.2. Introgression of Resistance Genes into Hexaploid Wheat via Synthetic Amphidiploid

From crosses between XueZao and the five SAW lines, 98 BC_2_F_1_ plants were obtained. Molecular marker analysis showed that 13 plants carried *YrU1*, 28 carried *Pm60b*, 10 carried *Pm60*, and six carried both *Pm60* and *YrU1* (Figure S1). All plants in the BC_1_F_2_ generation were genotyped using the markers *M-YrU1* and *M-Pm60*. Plants lacking the target resistance genes or exhibiting a selfed seed set rate of less than 50% were discarded. The BC_1_F_3_ plants were inoculated with *Pst* CYR34 and *Bgt* E09. The homozygous resistant BC_1_F_3_ lines were selected by phenotyping and marker analysis. From these, 11 families (Table 1) were selected for further development of hexaploid disease-resistant germplasm. The selected families were stable for disease resistance but still exhibited wild wheat traits to varying degrees, such as low selfed seed set, tall plant height, and brittle rachis, and need to break linkage drag further (Figure S2).

### 3.3. Successive Backcrossing and Selfing to Break Linkage Drag

**Figure 4:**
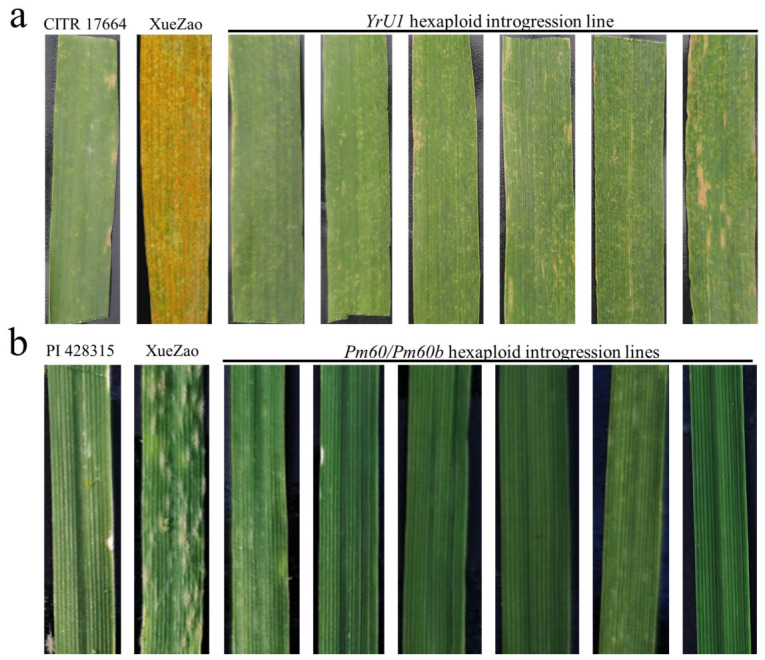
Comparison of resistance between disease-resistant introgression lines and resistant *Triticum urartu* parents. (**a**) Phenotypes of hexaploid introgression lines carrying *YrU1* after inoculation with mixed races of *Pst* (CYR32, CYR33, and CYR34); (**b**) phenotypes of hexaploid introgression lines carrying *Pm60* and *Pm60b* after inoculation with *Bgt* E09.

To break linkage drag, we continued to backcross the 11 selected introgression lines to the spring wheat cultivar Fielder to accelerate the breeding process. In the greenhouse, three successive crosses were performed using Fielder as the male parent with these 11 introgression lines, and at each generation, molecular marker detection and resistance evaluation were performed. Throughout the successive backcrossing process, we found that the hexaploid wheat genetic background did not compromise the resistance functions of *Pm60*, *Pm60b*, or *YrU1*; the backcross progenies exhibited resistance levels comparable to those of the original donor accessions (Figure 4). After two successive rounds of backcrossing, we selected 20 lines based on selfed seed set, spike morphology, and plant architecture for further advancement in the next generation. Ultimately, stable hexaploid wheat introgression lines carrying *Pm60*, *Pm60b*, *YrU1*, and *Pm60* + *YrU1* were obtained at the BC_2_F_4_ generation, which could be used as pre-breeding lines to improve wheat disease resistance.

### 3.4. Linkage Analysis of Resistance Genes and Phenotypes in the BC_1_F_1_ Generation

**Figure 5:**
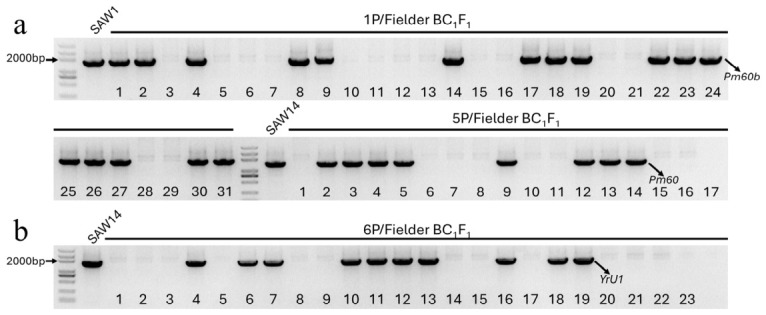
Marker detection of BC_1_F_1_ segregating population. (**a**) PCR-amplification of *M-Pm60* in 1P/Fielder BC_1_F_1_ and 5P/Fielder BC_1_F_1_. SAW1 and SAW14 were used as the positive control. (**b**) PCR-amplification of *M-YrU1* in 6P/Fielder BC_1_F_1_. SAW14 was used as the positive control. Numbers 1–31, 1–17, and 1–23 indicate lane numbers for three different families.

During the process of backcrossing, we performed linkage analysis of *Pm60*, *Pm60b*, and *YrU1* with phenotypes and investigated their segregation ratios in the BC_1_F_1_ generation. Among the 40 families examined, 34 fitted the expected 1:1 segregation ratio of resistant to susceptible plants, indicating that powdery mildew resistance and stripe rust resistance were each controlled by a single major gene in these populations. Moreover, the disease resistance phenotypes of individual plants were fully consistent with the genotyping results obtained using the *M-Pm60* and *M-YrU1* markers. Representative marker detection results and segregation patterns are shown in Figure 5 and Table 2.

### 3.5. Field Evaluation of Stable Resistant Introgression Lines Without Linkage Drag

**Figure 6:**
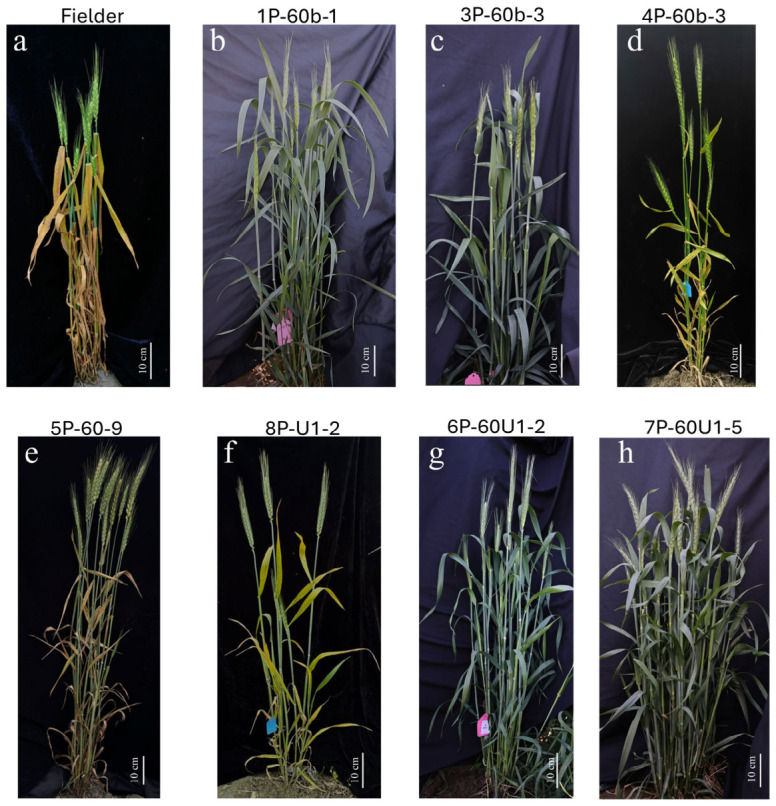
Plant morphology of resistant introgression lines. (**a**) Susceptible control Fielder. (**b**–**d**) *Pm60b* introgression lines. (**e**) *Pm60* introgression lines. (**f**) *YrU1* introgression lines. (**g**,**h**) *Pm60* + *YrU1* introgression lines. Scale bars, 10 cm.

**Figure 7:**
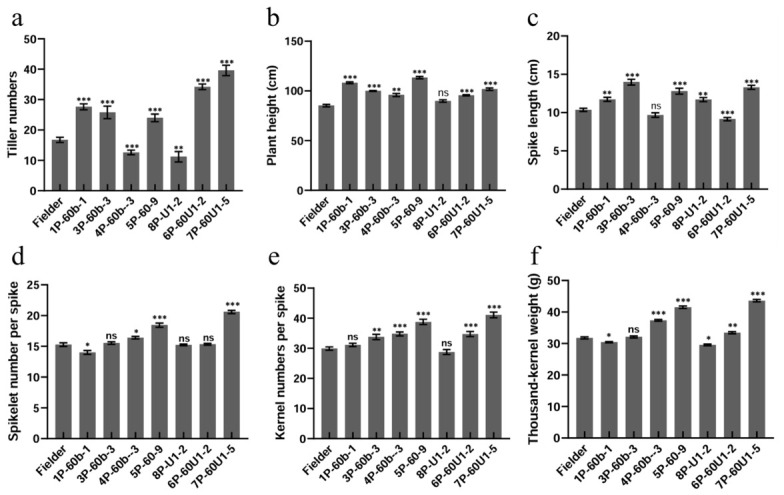
Comparison of key agronomic traits between resistant introgression lines and the recurrent parent Fielder. (**a**) Tiller number; (**b**) plant height; (**c**) spike length; (**d**) spikelet number per spike; (**e**) kernel number per spike; (**f**) thousand-kernel weight. Bars represent mean values (n = 10) and error bars indicate standard deviation (SD). Asterisks denote significant differences compared to Fielder (control) determined by Student’s *t*-test: * *p* < 0.05, ** *p* < 0.01, *** *p* < 0.001; ns = not significant.

After three generations of greenhouse screening and MAS, we obtained stable inherited lines with normal fertility, including 10 lines carrying *Pm60*, eight lines carrying *Pm60b*, 13 lines carrying *YrU1*, and eight lines carrying *Pm60* + *YrU1*. Despite multiple generations of backcrossing with hexaploid wheat, some resistant introgression lines still retained *T. urartu*-like phenotypes in plant architecture, spike morphology, and grain traits, likely due to suppressed recombination caused by local chromosomal differences and linkage drag in the genomic regions flanking the target genes *Pm60* and *YrU1*. Therefore, we selected 77 lines encompassing four resistance gene combinations under field conditions. These lines showed immunity-level resistance to the target diseases and no obvious adverse agronomic effects under the tested field conditions, and were designated as novel disease-resistant germplasm (Figure 6). During the 2024–2025 growing season, systematic evaluation of field agronomic traits was conducted on these novel disease-resistant germplasm lines (Figure 7; Table S4).

## 4. Discussion

As the progenitor of the A subgenome of common wheat, *T. urartu* represents an underutilized but important gene pool for disease resistance breeding. Compared with more distantly related wild species such as rye (*Secale cereale*) and *Dasypyrum villosum*, the high homology between *T. urartu* and the wheat A-genome facilitates homologous recombination [24], allowing rapid reduction in alien segments and elimination of linkage drag in fewer backcross generations. Earlier studies successfully transferred stem rust resistance genes *Sr21*, *Sr22*, and *Sr35* from *Triticum monococcum* (another A-genome donor) into common wheat using conventional crossing and backcrossing strategies [11,12,13]. These pioneering works demonstrated the feasibility of introgressing resistance genes from diploid A-genome species into hexaploid wheat. In recent years, several resistance genes have been cloned from *T. urartu*, including the powdery mildew resistance gene *Pm60* and its functional alleles *Pm60a* and *Pm60b*, as well as the stripe rust resistance gene *YrU1*. Notably, a single *T. urartu* accession can carry multiple resistance genes against different pathogens, which provides an ideal starting point for a “multi-resistance, multi-combination” pyramiding strategy. Pyramiding multiple resistance genes from the same donor into common wheat not only enables the resulting variety to resist multiple diseases but also greatly reduces the reliance on chemical fungicides. In the present study, *T. urartu* accessions carrying different *Pm60* alleles and *YrU1* were used to develop hexaploid wheat lines with resistance to powdery mildew, stripe rust, or both diseases.

In distant hybridization, reproductive barriers are the primary obstacle to gene introgression. Using tetraploid wheat as a bridge—first crossing it with *T. urartu* followed by chromosome doubling to create a synthetic amphidiploid (AABBA^u^A^u^) and then crossing this with common wheat—is an effective strategy to overcome this barrier. This strategy has been applied to introgress resistance genes from multiple wild relatives into wheat. For example, the stripe rust resistance gene *YrAS2388* [25] and the pre-harvest sprouting resistance gene *RSP* from *Aegilops tauschii* have been introduced into wheat via synthetic hexaploid wheat bridges and have become important resources for wheat improvement. The powdery mildew resistance gene *PmNCA6* [26] from *Triticum boeoticum* has also been introgressed through this pathway. Using this synthetic amphidiploid bridge approach, we introgressed *Pm60, Pm60b* and *YrU1* into a hexaploid wheat background, demonstrating the effectiveness of the “durum as a bridge” strategy for utilizing genes derived from *T. urartu*.

However, linkage drag frequently occurs during alien introgression, as donor segments may carry hitchhiking sequences. The introgression of rye chromosome 1R carrying *Pm8* and *Yr9* significantly enhanced wheat disease resistance and yield, but also caused minor quality defects due to linkage drag [27,28]. Similarly, *Pm13* from *Aegilops longissima* was transferred into wheat over 30 years ago but has not been widely used, largely due to linkage drag [16]. Therefore, when creating introgression lines carrying alien resistance genes, the potential impact of linkage drag must be fully recognized, and targeted measures, such as multi-generation backcrossing, must be taken to eliminate it.

Efficient selection methods are essential for introgressing novel genes from wild relatives into cultivated wheat. MAS enables accurate identification of target genes in early generations, preventing individuals lacking the desired genes from consuming breeding resources. However, marker-based selection alone is insufficient. Because alien gene expression may be affected by genetic background or position effects, multi-generation phenotypic evaluation is needed to confirm stable resistance [29]. Phenotypic evaluation also captures gene-by-environment interactions and provides direct evidence for selection decisions [30]. Combining MAS with phenotypic evaluation improves selection efficiency while ensuring functional integrity of the introgressed genes.

Eliminating or reducing linkage drag is central to practical use of introgressed genes. Multi-generation backcrossing is the primary means of reducing donor segment size, but the number of backcross generations must be balanced against breeding efficiency and background recovery. Our results support a phased strategy: in early generations, MAS for foreground selection to eliminate non-carriers or poor-fertility individuals, and in later generations, selection for agronomic traits such as selfed seed set, spike morphology, and plant architecture. This phased strategy—“marker selection in early generations, phenotypic selection in advanced generations”—maximizes breeding efficiency while minimizing linkage drag. Moreover, the homology between *T. urartu* and the wheat A-genome offers a natural advantage in accelerating the reduction in donor segments through homologous recombination. After multiple rounds of backcrossing and selfing, this study obtained stable hexaploid introgression lines carrying *Pm60*, *Pm60b*, *YrU1*, or *Pm60* + *YrU1* that showed immunity-level resistance to powdery mildew and stripe rust and no obvious adverse agronomic effects under the tested conditions. These lines can be used as donor parents to improve elite cultivars and to develop varieties with dual resistance and this technical pipeline can also be extended to other wild relatives and resistance genes.

## Figures and Tables

**Table 1:** Phenotypic investigation of initial introgression lines.

No.	Pedigree	Yellow Rust Response (IT)	Powdery Mildew Response (IT)	Resistance Gene	Self-Fertility Rate	Brittle Rachis
1P-3	(Mo75/PI 428215) × XueZao *^2^ F_3_	7	0	*Pm60b*	61.29%	No
1P-5	(Mo75/PI 428215) × XueZao *^2^ F_3_	8	0	*Pm60b*	53.53%	No
2P-1	(Mo75/PI 428315) × XueZao *^2^ F_3_	8	0	*Pm60b*	74.51%	No
3P-3	(Mo75/CITR 17664) × XueZao *^2^ F_3_	0	4	*YrU1*	58.41%	Yes
3P-17	(Mo75/CITR 17664) × XueZao *^2^ F_3_	0	4	*YrU1*	61.29%	No
4P-1	(Mo75/PI 428315) × XueZao *^2^ F_3_	8	0	*Pm60b*	60.64%	Yes
5P-7	(Mo75/CITR 17664) × XueZao *^2^ F_3_	8	0	*Pm60*	76.77%	No
6P-6	(Mo75/CITR 17664) × XueZao *^2^ F_3_	0	0	*YrU1* + *Pm60*	54.44%	No
6P-9	(Mo75/CITR 17664) × XueZao *^2^ F_3_	0	0	*YrU1* + *Pm60*	62.64%	No
7P-6	(Mo75/CITR 17664) × XueZao *^2^ F_3_	7	0	*Pm60*	61.97%	No
8P-3	(Mo75/CITR 17664) × XueZao *^2^ F_3_	0	0	*YrU1* + *Pm60*	76.54%	No

Note: *^2^ indicates two successive crosses with the corresponding male parent.

**Table 2:** Resistant and susceptible segregation in the BC_1_F_1_ population.

No.	Female (♀)	Male (♂)	Number of Seedlings		χ^2^_(1:1)_	*p*-Value
			Resistant	Susceptible		
1PF-BC_1_F_1_	(Mo75/PI 428215) × XueZao *^2^ F_3_/Fielder	Fielder	17	14	0.290	0.590
4PF-BC_1_F_1_	(Mo75/CITR 17664) × XueZao *^2^ F_3_/Fielder	Fielder	8	9	0.059	0.808
6PF-BC_1_F_1_	(Mo75/CITR 17664) × XueZao *^2^ F_3_/Fielder	Fielder	10	13	0.391	0.532

Note: *^2^ indicates two successive crosses with the corresponding male parent.

## Data Availability

The genotype data and the plant materials reported in this study are available upon request.

## Supplementary Materials

The following supporting information can be downloaded at: https://www.mdpi.com/article/10.3390/plants15121802/s1, Table S1: Information and disease resistance evaluation of *T. urartu* accessions; Table S2: Evaluation of powdery mildew resistance, leaf rust resistance, and self-fertility rate of synthetic amphidiploid wheat (SAW) lines; Table S3: Information of functional markers for *YrU1* and *Pm60*; Table S4: Investigation of agronomic traits in resistant introgression lines; Figure S1: Presence of resistance genes *YrU1*, *Pm60b*, and *Pm60* in primary introgression lines; Figure S2: Comparison of field plant architecture at the same growth stage between common hexaploid wheat and primary introgression lines. (a) Common hexaploid wheat; (b,c) primary introgression lines with obvious linkage drag.
