# Supplementary material for: Development of Hexaploid Wheat Germplasm with Resistance to Both Powdery Mildew and Stripe Rust by Introgression of Pm60 and YrU1 from Triticum urartu
Source: Plants (Basel). 2026 Jun 11;15(12):1802. doi: 10.3390/plants15121802 (PMC13306359; doi:10.3390/plants15121802)
Supplement: Supplementary file 1 [file plants-15-01802-s001.zip › plants-4333995-supplementary.pdf]

Table S1. Information and disease resistance evaluation of *T. urartu* accessions.

ID	Collection Site	Species	Yellow rust response (IT)	Powdery mildew response (IT)
PI 428215	Turkey	*T. urartu*	7	0
PI 428315	Lebanon	*T. urartu*	7	0
CITR 17664	Lebanon	*T. urartu*	0	0

Table S2. Evaluation of powdery mildew resistance, leaf rust resistance, and self-fertility rate of synthetic amphidiploid wheat (SAW) lines.

NO	Pedigree	Genome	Yellow rust resistance	Powdery mildew resistant	Self-fertility rate
SAW1	Mo75/PI 428215	$AABBA^uA^u$ (2*n*=6*x*=42)	6	0	22.86%
SAW2	Mo75/PI 428215	$AABBA^uA^u$ (2*n*=6*x*=42)	7	0	12.50%
SAW9	Mo75/PI 428315	$AABBA^uA^u$ (2*n*=6*x*=42)	6	0	32.35%
SAW10	Mo75/PI 428315	$AABBA^uA^u$ (2*n*=6*x*=42)	7	0	18.92%
SAW14	Mo75/CITR17664	$AABBA^uA^u$ (2*n*=6*x*=42)	1	0	26.67%

Table S3. Information of functional markers for *YrU1* and *Pm60*

Gene	Chromosome location	Marker name	Forward (F) and reverse (R) primer sequences (5' to 3')	Amplicon length
Pm60	7A	*M-Pm60*	F:CATTAACTTTGAGTTGTTGGA<br>R: CGGTGATCATACCAGAATTC	1551 bp
Pm60b	7A	*M-Pm60*	F:CATTAACTTTGAGTTGTTGGA<br>R:CGGTGATCATACCAGAATTC	1791 bp
YrU1	5A	*M-YrU1*	F: TGGGAAGATGCTCACAGAAGGTA<br>R:CTCAAACTCGGTCAAATTCGTAG	1738 bp

Table S4. Investigation of agronomic traits in resistant introgression lines

Resistant introgression lines	Tiller numbers	Plant height (cm)	Spike length (cm)	Flag leaf length (cm)	Flag leaf width (cm)	Spikelet number per spike	Kernel numbers per spike	Thousand-kernel weight (g)
Fielder	16.8±1.6	85.0±6.4	10.3±0.5	17.8±1.9	1.6±0.2	15.3±1.4	29.8±2.6	31.7 ± 2.3
1P-60b-1	27.5±2.5	108.1±5.7	11.6±1.5	22.7±3.7	1.7±0.1	14.0±1.6	31.0±2.6	30.4±1.2
3P-60b-3	25.2±4.8	99.9±3.8	13.9±2.1	18.3±2.1	1.6±0.1	15.5±1.0	33.6±3.3	32.0±2.4
4P-60b--3	12.5±1.5	96±7.6	9.7±1.0	11.0±0.7	1.3±0.2	16.3±1.2	34.6±3.0	37.3±0.9
5P-60-9	23.8±3.8	113.0±7.2	12.8±1.7	13.4±3.8	1.4±0.1	18.5±1.6	38.6±3.2	41.4±1.7
8P-U1-2	10.5±5.6	89.4±4.4	11.6±1.3	16.1±2.2	1.5±0.2	15.2±0.8	28.6±3.2	29.4±2.1
6P-60U1-2	34.0±3.4	95.3±4.1	9.1±0.9	21.6±2.0	1.6±0.1	15.3±1.0	34.6±3.6	33.4±2.0
7P-60U1-5	39.3±6.7	101.0±7.4	13.3±1.8	19.9±2.4	1.7±0.2	20.5±1.0	41.0±3.4	43.5±2.6

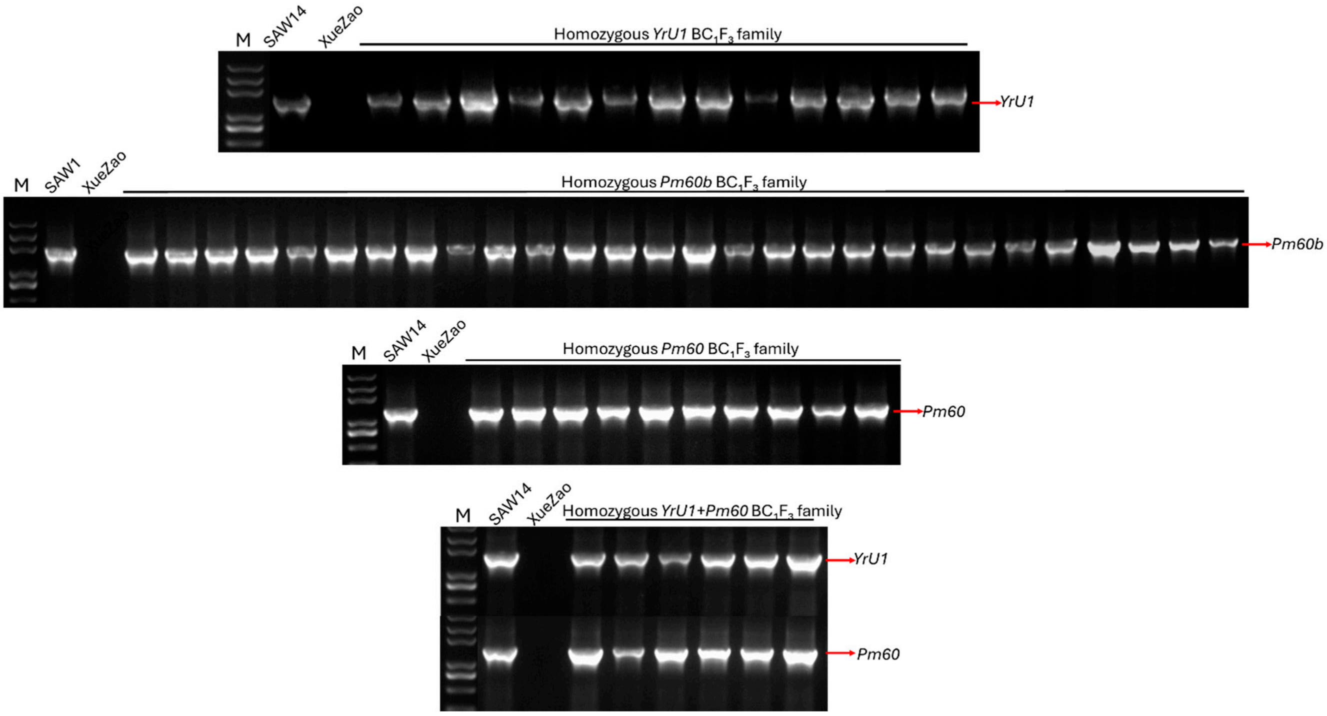

Figure S1. Presence of resistance genes *YrU1*, *Pm60b*, and *Pm60* in primary introgression lines.

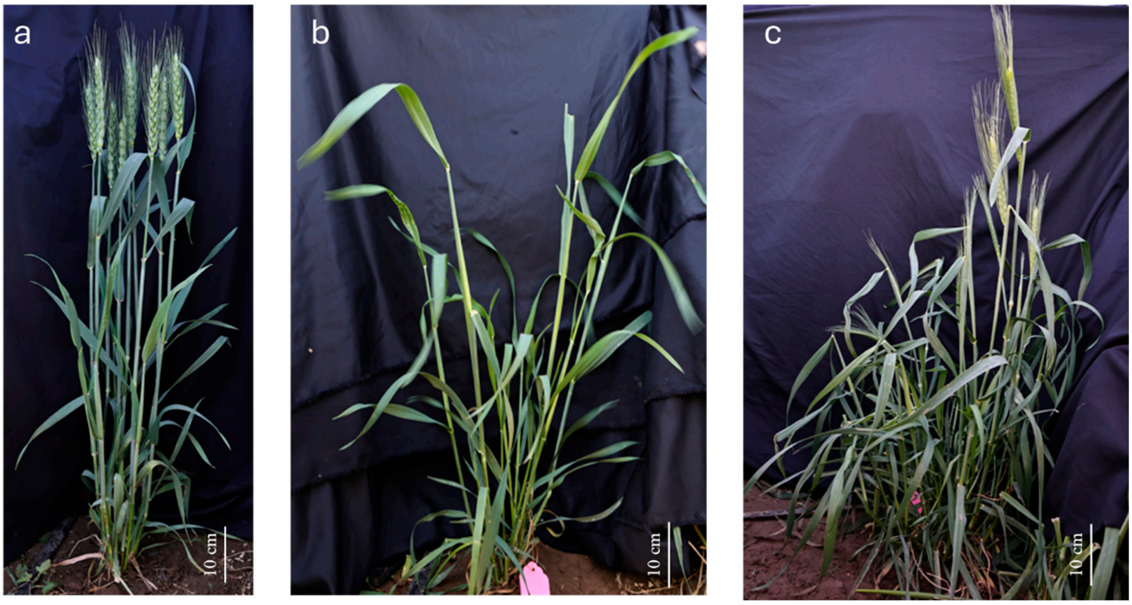

Figure S2. Comparison of field plant architecture at the same growth stage between common hexaploid wheat and primary introgression lines. a. Common hexaploid wheat; b, c primary introgression lines with obvious linkage drag.
